# Canine Cyanotoxin Poisonings in the United States (1920s–2012): Review of Suspected and Confirmed Cases from Three Data Sources

**DOI:** 10.3390/toxins5091597

**Published:** 2013-09-24

**Authors:** Lorraine C. Backer, Jan H. Landsberg, Melissa Miller, Kevin Keel, Tegwin K. Taylor

**Affiliations:** 1 National Center for Environmental Health, Centers for Disease Control and Prevention, 4770 Buford Highway NE, MS F-60, Chamblee, GA 30341, USA; 2 Fish and Wildlife Research Institute, Florida Fish and Wildlife Conservation Commission, 100 Eighth Avenue SE, St. Petersburg, FL 33701, USA; E-Mail: jan.landsberg@myfwc.com; 3 Marine Wildlife Veterinary Care and Research Center, Department of Fish and Wildlife, Office of Spill Prevention and Response, 1451 Shaffer Rd, Santa Cruz, CA 95060, USA; E-Mails: melissa.miller@wildlife.ca.gov (M.M.); tktdvm@gmail.com (T.K.T.); 4 School of Veterinary Medicine, University of California at Davis, Davis, CA 95616, USA; E-Mail: mkkeel@ucdavis.edu

**Keywords:** anatoxin, dog, canine, cyanotoxin, hepatotoxin, microcystin, neurotoxin poisoning, cyanobacteria, blue-green algae

## Abstract

Cyanobacteria (also called blue-green algae) are ubiquitous in aquatic environments. Some species produce potent toxins that can sicken or kill people, domestic animals, and wildlife. Dogs are particularly vulnerable to cyanotoxin poisoning because of their tendency to swim in and drink contaminated water during algal blooms or to ingestalgal mats.. Here, we summarize reports of suspected or confirmed canine cyanotoxin poisonings in the U.S. from three sources: (1) The Harmful Algal Bloom-related Illness Surveillance System (HABISS) of the National Center for Environmental Health (NCEH), Centers for Disease Control and Prevention (CDC); (2) Retrospective case files from a large, regional veterinary hospital in California; and (3) Publicly available scientific and medical manuscripts; written media; and web-based reports from pet owners, veterinarians, and other individuals. We identified 231 discreet cyanobacteria harmful algal bloom (cyanoHAB) events and 368 cases of cyanotoxinpoisoning associated with dogs throughout the U.S. between the late 1920s and 2012. The canine cyanotoxin poisoning events reviewed here likely represent a small fraction of cases that occur throughout the U.S. each year.

## 1. Introduction

Cyanobacteria, also called blue-green algae, are an ancient class of microorganisms found in all aquatic environments. Species within several genera produce potent toxins, known as cyanotoxins, including anatoxin-a, anatoxin-a(s), cylindrospermopsins, microcystins, nodularins, and saxitoxins, all of which can induce severe or fatal illness in animals and people (e.g., [1,2]). Mounting evidence indicates that global climate change, watershed degradation, and increased nutrient loading of freshwater systems are contributing to the increased frequency, severity, extent, and broader geographic distribution of harmful algal blooms (HABs) [3,4], including cyanobacteria HABs (cyanoHABs) [5]. Moreover, each year our desire to live along shorelines and our reliance on large surface waters for recreation and drinking water put more people and animals at risk for exposure to HABs and HAB-associated toxins.

Although cyanotoxins (including neurotoxins, hepatotoxins, and dermatologic toxins) are increasingly important and pervasive environmental pollutants, activities aimed at understanding the public health effects from exposure to these toxins are limited [6,7,8]. The potential for human and animal exposure to cyanotoxins in drinking and recreational waters has not been evaluated systematically, and we know little about public health effects from non-lethal toxin exposure [9]. 

Animals more commonly suffer fatal cyanotoxin poisoning than humans do because they are more likely to swim in and drink from ponds with active cyanobacterial blooms, even though the water may have a surface scum or bad smell [10]. Dogs, cats, domestic livestock, and wildlife (birds, mammals) have died from microcystin and anatoxin-a poisoning after drinking contaminated water or grooming cyanobacterial scum from their fur or feathers after swimming [11]. In fact, animals may sometimes seek out and eat dried cyanobacterial mats or crusts, which may contain toxins [11]. The impacts of these cyanotoxins on domestic and wild animals are significantly under-recognized because many cases are misdiagnosed, few cases are biochemically confirmed, and even fewer are reported in the scientific literature or to animal health surveillance systems [12].

Cyanotoxins can be inhaled and ingested, and exposure to these toxins can induce acute, subacute, or chronic poisoning effects in animals and people [7,13,14,15,16,17]. Efforts to obtain a more accurate assessment of the extent of canine intoxication by cyanotoxins and to identify high-risk areas for pet exposure may also facilitate characterizing human health risks and protect public health. In our report, “event” refers to a cyanoHAB and “case” refers to an ill or dead dog.

## 2. Results and Discussion

### 2.1. Harmful Algal Bloom-Related Illness Surveillance System (HABISS)

Between 2007 and 2011, Departments of Health and/or Environment from 13 states (including 9 states funded by NCEH: Florida, Iowa, Maryland, Minnesota, New York, North Carolina, Oregon, Virginia, Wisconsin and 4 additional states: California, Kansas, Montana, Texas) reported 67 suspected or confirmed cases of canine intoxications associated with HABs. Of these 67 cases, 58 (87%) followed exposure to fresh waters and 1 (1%) followed exposure to marine waters. The exposure source was unknown for the remaining 9 cases (13%). Among the cases, exposure was reported as inhalation for 9 (13%), ingestion for 6 (9%), dermal contact plus ingestion (*i.e.*, swimming, with accompanying ingestion of algae/toxins from swallowing water and/or licking algae off fur) for 36 (54%), and unknown for 16 (24%). Gastrointestinal symptoms, including vomiting and diarrhea, affected 29 dogs (43%). Other symptoms included lethargy in 12 cases (18%) and neurologic signs, including stumbling or change in behavior in 6 cases (9%).

Thirty-eight (58%) of the canine intoxications were fatal. Of these, 12 (32%) were attributed to anatoxin poisoning, 3 (8%) to microcystin poisoning, and 5 (13%) to exposure to an unspecified cyanotoxin. One dog died following exposure to brevetoxins produced by the marine dinoflagellate *Karenia brevis*. The specific cause of death was unknown for the remaining 17 dogs (45%). After further investigation, 3 (4%) of the 67 reported cases were categorized as not likely HAB-related but with the cause unidentified. In summary, of the 67 cases of canine intoxication associated with HABs reported in HABISS, 63 were possibly or confirmed to be associated with freshwater cyanoHABs. HABISS represented the first attempt to monitor human and animal health events simultaneously with associated HAB events. Although biased toward states funded to report case activity by NCEH, the system identified 20 canine deaths following cyanotoxin exposure. In addition to conducting HAB-related morbidity and mortality surveillance, funded states produced cyanoHAB-related educational content, and many made this information available on public health websites (see examples in Table 1).

**Table 1:** Examples of HAB-related information available on the world wide web.

Website address	Owner	Date of access
**State Resources**		
http://myfwc.com/research/redtide/task-force/reports-presentations/resource-guide/	Florida Fish and Wildlife Conservation Commission	3 May 13
http://www.kdheks.gov/algae-illness/index.htm	Kansas Department of Health and Environment	3 May 13
http://www.deq.virginia.gov/Programs/Water/WaterQualityInformationTMDLs/WaterQualityMonitoring/VirginiaHarmfulAlgalBloomTaskForce.aspx	Virginia Department of Environmental Quality	3 May 13
**Federal Agency Resources**		
http://www.cdc.gov/nceh/hsb/hab/default.htm	Centers for Disease Control and Prevention	3 May 13
http://www.epa.gov/gmpo/habpage.html	U.S. Environmental Protection Agency	3 May 13
http://pubs.usgs.gov/fs/2006/3147/pdf/FS2006_3147.pdf	U.S. Geological Survey	3 May 13
**Private Entity Resources**		
http://www.mote.org/index.php?src=faq&refno=142&category=Florida%20red%20tide	Mote Marine Laboratory	3 May 13
http://www.whoi.edu/redtide/	Woods Hole Oceanographic Institute	3 May 13

### 2.2. Suspected and Confirmed Canine CyanoHAB Intoxication Cases from the Veterinary Medical Teaching Hospital (VMTH) Necropsy and Biopsy Case Records, University of California, Davis

We searched VMTH canine biopsy and necropsy accessions between 1984 and 2012 for cases of acute hepatotoxicity or acute death that could be compatible with cyanotoxin poisoning in dogs. These cases are not compatible with the typical presentations expected from infection with waterborne microbes. We identified 71 cases that met study selection criteria, including 5 dogs (7%) with confirmed or strongly suspected mushroom intoxication, 2 dogs (3%) with confirmed or suspected anatoxin-a poisoning, and 43 dogs (61%) with a moderate to high possibility of microcystin intoxication based on the clinical presentation, lesion description, listed differential diagnoses, pathologist comments, and diagnostic test results. For the remaining 21 dogs (30%), the cause of death included factors other than cyanotoxins or poison mushrooms, such as anti-inflammatory or anti-seizure medications; thus, we exclude these cases from further review.

Interestingly, necropsy or biopsy accession records were also identified for 4 domestic cats with acute hepatic necrosis through the same keyword-based, VMTH database search. Mushroom poisoning had been biochemically confirmed for 1 cat, microcystin poisoning was suspected for a second cat, and no specific cause of hepatotoxicity was identified for the 2 remaining cats.

Based on our case-coding criteria described in Section 3.4, we considered the likelihood of exposure to microcystin or another hepatotoxic cyanotoxin to be moderate for 14 dogs (31%), and high or confirmed for 29 dogs (64%). However, significant overlap of hepatic lesions and clinical signs between microcystin intoxication and mushroom poisoning was noted, both conditions were commonly listed as differential diagnoses, and it would not be possible to definitively distinguish between the two conditions without testing samples, such as that of cryopreserved liver, that are no longer available. Because significant overlap exists between clinical signs and lesions ascribed to microcystin intoxication and mushroom poisoning and because toxin testing had been performed for only a few cases, it is possible that some cases of mushroom poisoning were misclassified as microcystin cases in the current study. Future studies could attempt immunohistochemical testing of archival paraffin blocks for microcystin and amanitin, where available, to help distinguish between these poisonings.

Mushroom poisoning was biochemically confirmed for 3 dogs and was strongly suspected for 2 additional dogs. These 5 cases consisted of 3 males and 2 females. Their deaths spanned 2001 through 2010, and all were from central or Western California (Santa Cruz, Contra Costa, Napa and Yolo Counties). Affected breeds included a Chesapeake Bay Retriever, a Beagle/Pug mix, a Labrador/Poodle mix, a Welsh Corgi, and a Boston Terrier. All animals presented for necropsy following 1–3 days of illness during the months of May (1 dog), June, (2 dogs), August (1 dog) and September (1 dog). The age of affected dogs ranged from 3 months to 3 years, but, interestingly, 4 of the 5 dogs with strongly suspected or confirmed mushroom poisoning were ≤5 months old.

Cyanotoxin poisoning was biochemically confirmed from postmortem samples for 2 for the 45 dogs (4%) with suspected or confirmed cyanotoxin poisoning, including 1 of 2 dogs that died following acute anatoxin poisoning, and 1 dog that died from microcystin intoxication. Both canine anatoxin cases involved dogs from the same household. The dogs died peracutely (within 20–30 minutes of onset of illness) following exposure to cyanobacteria in a backyard pond. One of the dogs was 6 months old, and the report did not note the age of the second dog. Additional dogs in the household that did not have access to the pond were unaffected, and biochemical testing identified anatoxin-a in kidney collected during necropsy from the 6-month-old dog. We did not find any other cases of fatal canine anatoxin poisoning in the database.

Of the 43 dogs with a moderate to high probability of microcystin intoxication, a wide range of dog breeds was noted, including mixed-breed dogs (8 dogs; 19%), Golden Retrievers (6 dogs; 14%), Labrador Retrievers (3 dogs; 7%), German Shepherds, Pomeranians, Rottweilers and Poodles (2 dogs each) and numerous other dog breeds. The affected dog breed was not noted for 2 cases. Twenty of the dogs were intact or castrated males, and 23 were intact or spayed females. The age range of affected dogs encompassed <3 months to 12 years, but 79% of dogs with suspected or confirmed microcystin intoxication were ≥1 year old, in sharp contrast with the mushroom poisoning cases above. The majority of suspected canine microcystin poisonings were from Northern, Central, or coastal California, including Marin County (7 cases); Sacramento and Yolo Counties (4 cases each); Alameda, San Joaquin, El Dorado and Placer Counties (3 cases each); Napa, Contra Costa, and San Francisco Counties (2 cases each); and 1 case each from Amador, Calaveras, Fresno, Nevada, San Bernardino, San Mateo, Shasta, Solano, Stanislaus, and Yuba Counties.

The reported duration of illness ranged from <1 day to 6 weeks. However, the duration of illness was between 1 and 7 days for 74% of cases, and was ≥3 days for 60% of cases, in sharp contrast with the shorter duration of illness reported for the anatoxin and mushroom poisoning cases described above. The report did not include the duration of illness for one of the 43 suspect microcystin cases. The majority of affected dogs (86%) were presented for necropsy or biopsy in good or obese nutritional condition; however, nutritional condition was not noted for 7 dogs.

The number of microcystin-suspect cases examined by pathologists each month was highest in November (7 dogs) and June (6 dogs), followed by January and May (5 dogs each), April, July and August (4 dogs each), October (3 dogs), September and December (2 dogs each), and March (1 dog). Cases appeared to be more common over the warmer late spring, summer and fall months (April through November), and the early wet season in California (November through January). The only month when VMTH Pathologists did not identify a microcystin poisoning-suspect dog was February. The mild climate that exists throughout much of California may explain the lack of a strong seasonal case distribution, in contrast with areas of the United States that experience colder winters and a shorter warm season. Suspected or confirmed microcystin intoxication cases spanned from 1987 through 2011, with the highest number of cases observed in 2009 (5 cases), 2005 (4 cases) and 2000, 2001 and 2002 (3 cases each). The yearly proportion of suspect or confirmed microcystin cases from 1987 through 1999 was 1.08/year, compared with 2.49/year for the period from 2000–2011. It is unknown whether this apparent increase in case frequency over the latter half of the case accession period is due to an increased prevalence of microcystin intoxication in dogs, enhanced case recognition, random chance, or a combination of factors.

Review of retrospective case records from the VMTH, a large regional teaching hospital, provides a snapshot of the range of cases presenting to local veterinarians throughout Northern and Central California, the area of the state that this facility most heavily supports. However, because the VMTH is primarily a referral hospital, cases of acute toxicosis and acute hepatic failure in dogs are probably far more common than these data indicate, with many dogs dying in local homes and veterinary clinics without referral to the VMTH. Cyanotoxin poisoning cases are also under-reported because cyanotoxin tests are expensive, access to testing is limited in the state, and diagnosis may not be a priority for the owner after the dog has died. Finally, based on the misperception that no specific therapies exist for treatment of microcystin intoxication and mushroom poisoning, some pet owners and veterinarians might elect to euthanize suspect cases or provide limited supportive care without referral. However, there is increasing evidence that simple, cost-effective treatment modalities for microcystin and/or mushroom-poisoned animals could enhance the likelihood of recovery and shorten hospitalization time [18,19,20,21,22].

Time or cost constraints and client or veterinarian perceptions regarding potential environmental health risks can also limit the range of diagnoses that are considered. For example, cyanotoxin-associated dog deaths could erroneously be attributed to mushroom poisoning (and *vice-versa*) if a full history regarding recent outside activities such as swimming, camping, or cyanoHAB contact is not elicited from the owners. Interestingly, among the 71 dogs described above, we found that the differential diagnosis included mushroom intoxication more commonly for cases where the presentation and lesions could be consistent with either microcystin intoxication or mushroom poisoning, even when the report did not include prior history of mushroom consumption.

Veterinarians and pet owners may also assess risks for cyanoHAB exposure based on the affected dog breeds or for dogs living in specific settings. These assumptions may also influence the range of diagnoses that are considered. For example, veterinarians might assume that large, water-loving dog breeds such as Retrievers are more likely to encounter cyanotoxins than smaller dogs; or that urban-dwelling dogs are unlikely to encounter cyanotoxins in their local environment. Although sporting breeds such as Labrador and Golden Retrievers appear to be at slightly higher risk of cyanotoxin poisoning, we found that a wide range of dog breeds, including Poodles, Dachshunds and toy breeds are at risk of encountering cyanotoxins in lakes, rivers, backyard ponds and urban and residential water bodies. These water bodies tend to be shallow, stagnant and warm during the summer and fall months and can accumulate high levels of nutrients through runoff from nearby yards and gardens, providing ideal conditions for development of toxic cyanobacterial blooms.

It is also important to consider that the volume of published literature concerning a given environmental toxin shapes veterinary perceptions. For example, in a recent newsletter for Pacific Veterinary Specialists, an article about managing tremors and seizures mentioned mushroom toxicity, but not algal toxins, as a possible cause [23]. Mushroom poisoning appears to be a default diagnosis at present, when in fact the lesions of mushroom intoxication and microcystin poisoning are indistinguishable without testing. In the current study, only one of the 45 dogs (2%) examined at the VMTH between 1984 and 2011 with suspected or confirmed cyanotoxin poisoning was reported in the published scientific literature [24]. While microcystin intoxication was commonly considered as a differential diagnosis, biochemical testing to confirm cyanoHAB exposure was rarely performed (4% of cases), even at this large, regional veterinary care facility. Finally, veterinarians are often the first experts to recognize and respond to cyanoHAB events in communities through clinical care of microcystin-poisoned animals. The evidence that veterinarians are the first experts to recognize and respond to these events indicates that efficient information sharing among veterinarians, local public health officials, and water resource managers about suspected or confirmed cyanoHAB intoxication cases could help prevent additional animal poisonings and provide the warnings needed to prevent human poisonings.

### 2.3. Historical Reports from a Review of Scientific Publications, Media, and Other Electronically Available Sources

The earliest report (late 1920s) of a cyanoHAB event affecting dogs in the U.S. was found in a California Department of Water Resources [25] publication. A dog became ill after drinking water and swimming in Clear Lake in California during an algal bloom and then licking a thick coating of algae from its coat. In 1944, an *Anabaena* bloom in a lake in the Okoboji chain of lakes in Iowa was blown onshore and caused fatal poisoning of pigs and at least one dog that drank from the lake [26]. Other reports of canine cyanotoxin poisonings occurred sporadically until the mid-1970s, when newspapers and the scientific and medical literature reported dog deaths following exposure to cyanoHABs almost yearly.

Between the late 1920s and August 2012, media reports, state and federal agency reports and published scientific and medical literature described 115 cyanoHAB-related events involving 260 dogs; 215 (83%) of the dogs died and 45 (17%) became ill and then recovered (Table 2). 

**Table 2:** Summary of historical reports of canine poisonings from cyanotoxins in the U.S. identified through a review of scientific publications, media, and other electronically available sources.

#	Year	State	Reported Exposure	Cyanotoxin	# Dead	Breed ^1^	#Sick	Breed ^1^	Ref
1	late 1920s	CA			0		1		[25]
2	1944	IA	*Anabaena flos-aquae*		1				[26,27,28,29]
3	1948	MN	*A. lemmermannii*		2				[26]
4	1948	MN	*Microcystis aeruginosa*		2				[26,29]
5	1948	IA	*Anabaena flosaquae*		2				[26,28,29]
6	1952	IA	*Anabaena flos-aquae*		15				[26,28,30,31]
7	1969	FL	*Microcystis aeruginosa*^2^		1	German Shepherd			[32]
8	1976	WA	*Anabaena flos-aquae*		4		7		[33,34,35,36]
9	1977	MT	*Anabaena flos-aquae*	Anatoxin-a	8				[37,38,39]
10	1977	WA	*Anabaena flos-aquae*		1				[40]
11	1978	WA	*Anabaena flos-aquae*		0		1	German Shepherd	[34]
12	1979	PA	*Anabaena*		0		1		[41]
13	1980	MT	Cyanobacteria		2				[42]
14	1981	ID	*Anabaena flos-aquae*	Anatoxin-a	2				[43,44,45]
15	1982	WA	*Anabaena flos-aquae*		2	Black Labrador (2)	1		[36,44]
16	1985	WI			2	Collie, Labrador	1		[46,47]
17	1985	SD	*Anabaena flos-aquae*	Anatoxin-a(s)	9	German Shepherd			[48]
18	1985	SD	*Anabaena flos-aquae*	Anatoxin-a(s)	5				[48]
19	1986	WI	Cyanobacteria		3				[49]
20	1986	NJ	Cyanobacteria		1				[50]
21	1989	WI	Cyanobacteria		0		2		[51]
22	1989–1990	WA	*Anabaena flos-aquae*		1		5		[52,53,54]
23	1990s	UT	Cyanobacteria		2				[55]
24	1990	MS	Blue-green algae		14				[56,57]
25	1990	IN	*Anabaena flos-aquae*	Anatoxin-a	2				[58]
26	1991	SD	Blue-green algae		1				[59]
27	1991	OR	*Anabaena*		5	English Springer Spaniels (2), Cocker Spaniel			[60,61,62]
28	1991	CA	*Microcystis aeruginosa*		1	Golden Retriever			[24]
29	1997	WA	Cyanobacteria		1	Brittany Spaniel			[63]
30	1997	CA	Blue-green algae		1	Field Spaniel			[64]
31	1997	WA	*Microcystis aeruginosa*	Microcystins	1	Golden Retriever			[65,66]
32	1998	MN	Blue-green algae		1				[67]
33	1998	MD	*Microcystis aeruginosa^2^*		2				[68]
34	1998	MA	*Anabaena* sp.		2		2	Black Labrador mix	[69,70]
35	1999	NY		Anatoxin-a ^2^	2	Black Labrador-Golden Retriever mix, Chocolate Labrador			[71,72,73,74]
36	1999	VT	Cyanobacteria		2	Labrador Retriever			[72,75]
37	1999	ID	Cyanobacteria		6				[76,77]
38	1999	ID		Cyanotoxin	1				[78,79]
39	2000	NY	Blue-green algae	Anatoxin-a	2				[73]
40	2000	ID	Blue-green algae		3	Chocolate Labrador Retriever			[80,81,82]
41	2000–2001	OR	Cyanobacteria		2				[36]
42	2001	CA			5	Australian Shepherd-English Setter mix, Mutt			[36,83,84]
43	2002	NM	*Lyngbya* (“Mermaid’s hair”)	Neosaxitoxin	0		1	Golden Retriever	[85,86]
44	2002	NM	*Lyngbya* (“Mermaid’s hair”)		1				[85]
45	2002	CA	*Anabaena*, *Lyngbya, Planktothrix*	Anatoxin-a	1				[83,87]
46	2002	CA	*Anabaena*, *Lyngbya, Planktothrix*	Anatoxin-a	2				[83,87]
47	2002	VT		Microcystins, Anatoxin-a	1				[73,88,89]
48	2003	WI	Blue-green algae ^2^		1				[90]
49	2003	SD			2	Chocolate Labrador			[91]
50	2004	NE	*Anabaena*	Microcystins	3				[92,93,94,95]
51	2004	NE	Cyanobacteria		3	Yellow Labrador, Sheep-dog, Australian Shepherd			[95,96,97]
52	2004	NY	*Anabaena, Microcystis*	Microcystins, Anatoxin-a	1	Labrador Retriever	1		[98,99,100]
53	2004	WI	*Anabaena, Microcystis*, *Aphanizomenon*		1	Labrador Retriever	1		[101,102,103]
54	2004	MN	Cyanobacteria		1				[104]
55	2004	CA		Cyanotoxin ^2^	1				[83]
56	2004	ID	Cyanobacteria		1	Labrador	1		[105]
57	2004	MN	*Microcystis* ^2^		1				[106]
58	2004	WA			1	Chocolate Labrador-Doberman mix			[107,108]
59	2005	WA	*Anabaena*		3				[36,108]
60	2005	WI			1				[109]
61	2005	IA	Cyanobacteria		0		1		[110]
62	2006	NE	*Anabaena* sp.	Anatoxin-a	2	German Shorthair Pointer			[111,112]
63	2006	WA	*Anabaena, Microcystis*, *Aphanizomenon*	Anatoxin-a	2		1		[36,113,114]
64	2006	ID	Cyanobacteria		0		1		[115]
65	2007	MN	Cyanobacteria		1	Yellow Labrador			[116,117,118]
66	2007	MI		Microcystin ^2^	1	Border collie			[119,120]
67	2007	MN	Cyanobacteria		1				[121]
68	2007	KS			3		1		[122]
69	2007	MT	Toxic blue-green algae		1				[123]
70	2007	MT	Toxic blue-green algae		0		1		[123]
71	2007	WA	Cyanobacteria		2	Hunting dogs (2)			[124]
72	2007	MN	Cyanobacteria		3	Golden Retriever, Cocker Spaniel, Bernese Mountain Dog			[121,125]
73	2007	NM	Blue-green algae		1	Jack Russell Terrier			[126,127,128]
74	2007	WI	Blue-green algae		1	Brittany Spaniel			[129]
75	2007	WA		Anatoxin-a ^2^	1				[114]
76	2008	KS	Blue-green algae		1	Australian Shepherd	2	Australian Shepherd, Labradoodle	[130]
77	2008	MT	Cyanobacteria		1				[131]
78	2008	WA			2	Labrador Retriever (2)			[114]
79	2008	WA			2		1		[114]
80	2008	MN	Cyanobacteria		3				[132]
81	2009	WI	Cyanobacteria		2				[133]
82	2009	WI	Cyanobacteria		1	Australian Terrier			[134]
83	2009	WA	Blue-green algae		0		2		[135]
84	2009	NM			2	Pit Bull (2)			[136,137]
85	2009	ND	*Microcystis*		1				[138]
86	2009	CA	Cyanobacteria		1	Blue Heeler cross			[139]
87	2009	CA	Blue-green algae		1				[140]
88	2009	OR		Anatoxin-a	4	Border Collie, Labrador mix, Husky, Blue Heeler			[141,142,143,144,145]
89	2009	MN			1	Rat Terrier			[146]
90	2009	TX			0		2		[147]
91	2009	NM			1	Labrador			[136]
92	2009	MN	Blue-green algae		1	Black Labrador			[148,149,150,151]
93	2009	WA	Blue-green algae ^2^		1	Labrador Retriever			[152]
94	2009	IN	Blue-green algae ^2^		1	Golden Retriever			[153]
95	2010	WI			1				[154]
96	2010	WI	Blue-green algae		1		1		[154]
97	2010	OH	Cyanobacteria		3	Black Labrador Retriever, Rat Terrier, Golden Retriever	1	Cairn Terrier	[155,156]
98	2010	OH			2				[157]
99	2010	ND	3 types toxic algae		1				[158]
100	2010	OR	Blue-green algae	Anatoxin-a	1	Labrador			[145,159]
101	2010	NY	Blue-green algae		1				[160]
102	2010	MT	Blue-green algae		1	Australian Shepherd			[131,161]
103	2011	MA	Blue-green algae		0		1		[162]
104	2011	OR	Algal scum		1	Jack Russell Terrier			[163]
105	2011	KS	Cyanobacteria		3	German Shepherd?	1		[164]
106	2011	OR	Green scum		1	Springer Spaniel			[165]
107	2011	OH	Blue green algae	Negative for microcystin	1	Labrador Retriever mix			[166,167]
108	2011	KS	*Microcystis*		1	Briard			[168]
109	2012	OK	Blue green algae		2				[169]
110	2012	WI			1				[170]
111	2012	IN	Blue green algae		2	Short-Haired Pointer, Labrador mix	2		[171,172]
112	2012	NY			2				[173]
113	2012	CA			0		1		[174]
114	unknown	CA			1	Pit Bull			[175]
115	unknown	CA			0		1		[176]
Total					215		45		

^1^ Not all corresponding breed data for the number of dead or ill dogs were available; ^2^ highly suspected based on circumstantial evidence.

**Figure 1:**
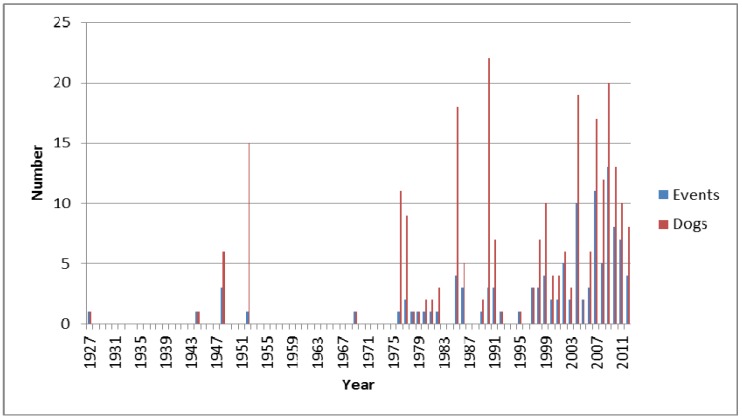
Number of reported cyanobacteria harmful algal blooms (cyanoHAB) events between the late 1920s and 2012 that were associated with dog morbidity and mortality, and number of dogs involved in these events (identified through review of media reports and historical literature).

The events spanned 27 states (California, Florida, Idaho, Indiana, Iowa, Kansas, Maryland, Massachusetts, Michigan, Minnesota, Mississippi, Montana, Nebraska, New Jersey, New Mexico, New York, North Dakota, Ohio, Oklahoma, Oregon, Pennsylvania, South Dakota, Texas, Utah, Vermont, Washington, and Wisconsin). The highest number of reports were from Washington (*n* = 16), then California (*n* = 12), followed by Minnesota (*n* = 11). Figure 1 summarizes the temporal distribution of reported events as well as the number of dogs involved. Most years prior to the late 1970s experienced 0–4 events involving dogs exposed to cyanoHABs and/or cyanotoxins each year. At least 5 events/year were reported during 2002, 2004, and between 2007 and 2011. The highest number of separate cyanoHAB events affecting dogs was reported in 2009 (*n* = 14), and the highest number of dogs affected by all known cyanoHAB events during a single year was reported in 1990 (*n* = 15). The number of annual HAB events associated with reports of adverse health effects for dogs and the number of affected dogs has increased substantially, beginning in the mid-1970s. This may result from increased prevalence of severe cyanobacterial blooms, enhanced public awareness, enhanced cyanoHAB detection and/or broader recognition of possible negative impacts from global climate change. Potential contributing factors include increasing water surface temperatures [177], enhanced communication technology, improved analytical methods and increased awareness of potential public health and ecological health threats from HABs.

The affected dog breed was noted in 66 (31%) of the 215 fatal poisonings. Of the events in which dog breed was identified, the most commonly reported breed was the Labrador Retriever (19 cases, 29%). Other breeds with >1 case of fatal poisoning included Golden Retriever (5 cases, 8%); Australian Shepherd, German Shepherd, and American Pit Bull (3 cases, 5% each); Border Collie, Brittany Spaniel, Cocker Spaniel (variety unknown), English Springer Spaniel, German Shorthaired Pointer, Jack Russell Terrier, and Rat Terrier (2 cases, 3% each). Breeds with a single case of fatal poisoning included Australian Terrier, Bernese Mountain Dog, Blue Heeler, Briard, Collie, Field Spaniel, Husky, Sheepdog, and Springer Spaniel (variety unknown). Two dogs were identified only as hunting dogs. Eight (12%) of the identified dog breeds with fatal poisonings were mixed breeds. In non-fatal cases, affected breeds included Australian Shepherd, Cairn Terrier, German Shepherd, Golden Retriever, and two mixed breeds. Clinical signs and symptoms observed in the dogs included severe diarrhea, vomiting, loss of motor coordination, restlessness, weakness, deep breathing, paralysis, and convulsions [31,48].

We identified 115 poisoning events from our search of which 102 were fatal. 39 were attributed to exposure to a specific cyanobacterial species or genus, to mixed genera or toxins, or to a specific cyanotoxin. Of these, 22 fatal poisoning events (58%) involving 76 dogs were attributed to *Anabaena* spp. and/or anatoxin-a or anatoxin-a(s); 10 events (26%) involving 14 dogs were attributed to exposure to *Microcystis*, *Anabaena* and/or microcystins; 6 events (16%) involving 8 dogs were attributed to exposure to mixed blooms of *Anabaena*, *Aphanizomenon*, and *Microcystis* and anatoxins/microcystins, and 1 event was attributed to *Lyngbya* (possibly *L. wollei* [178]) and the neurotoxin neosaxitoxin. In 44 events (76 dogs), dogs swam in or drank water with a visible bloom or surface scum and subsequently experienced clinical signs of anatoxin or microcystin poisoning. For the remaining fatal poisoning events (37 events, 49 dogs), the exposures were reported as swimming in or drinking water from a lake or reservoir and were followed by the appearance of clinical signs and symptoms consistent with anatoxin or microcystin poisoning. The majority of the 115 poisoning events occurred in lakes (69 cases, 60%) with 15 events in rivers (California [6], Oregon [5], Idaho [2], Maryland [1], Texas [1]), 12 in reservoirs, 12 in ponds, and 7 in unidentified water bodies (data not shown). 

There were 30 non-fatal poisoning events involving 45 dogs; 16 of these accompanied fatal events. Of the non-fatal events, 5 (17%) were attributed to exposure to *Anabaena* spp. or anatoxin; 3 were attributed to mixed blooms of *Anabaena*, *Aphanizomenon*, and *Microcystis* and anatoxins/microcystins; 1 event was attributed to *Lyngbya*, and no events were attributed to exposure to *Microcystis* spp. or microcystins. Of these non-fatal cases that were attributed to cyanotoxicosis, 6 (75%) co-occurred with fatal cases. Cyanobacteria/blue-green algae exposure was noted in 12 events (43%) involving 16 dogs.

Although many early reports identified the specific cyanobacterium responsible for animal poisonings, investigators assigned toxin names and developed analytical methods much later. For example, Carmichael *et al*. [179] identified *Anabaena flos-aquae* as the source of a neuromuscular toxin or toxins, known then as “very fast death factor,” in early reports describing animal deaths. In 1977, Devlin *et al*. [180] published a method to isolate the toxin (now called anatoxin-a) from cyanobacteria. In the same year, Carmichael [39] isolated anatoxin-a from *A. flos-aquae* collected from Hegben Reservoir, Montana, U.S., after 8 dogs and 30 cattle died soon after drinking water from the reservoir. While the ability to detect and quantify cyanotoxins is critical for assessing exposure, analytical test results may not be definitive. For example, mixed cyanobacteria species with several cyanotoxins (anatoxins and microcystins) were detected in bloom samples associated with a canine poisoning event; however, the mouse bioassay indicated acute neurotoxicosis suggestive of anatoxin-a, rather than microcystin, poisoning [48]. Over time, reports of analytical testing for cyanotoxin concentrations in water or stomach contents increased but did not replace the clinical history of antemortem exposure to cyanobacteria or presumed algal toxin as an important diagnostic indicator of the cause of death. Interestingly, in these reports, temporospatial association of the onset of dog illness with cyanobacterial blooms was considered the least specific diagnostic indicator, and biochemical detection of biotoxin in stomach contents was the most specific indicator of cyanotoxin exposure.

### 2.4. Dogs, CyanoHABs, and Public Health

Table 3 shows a summary of the number of events and cyanoHAB-related dog deaths identified by the three datasets. Of the 115 reports captured by the media search, 11 (10%) were also captured in HABISS. Of 11 cases in the VMTH dataset that occurred in the 2007–2011 timeframe, none was duplicated in HABISS and one was duplicated in the historical reports.

Using the above three data sources, we identified 230 discrete cyanoHAB events, and 367 cases of suspected or confirmed canine cyanotoxin poisoning in the U.S. between the late 1920s and 2012. Each dataset provides unique perspectives. The media reports emphasize unusual acute events or ongoing cyanoHAB events in particular communities. While they may not include medical details, these reports help identify water bodies that have historically supported toxin-producing cyanoHABs, and water bodies with recurrent or potentially worsening cyanoHAB issues. This information could facilitate predictions of cyanoHAB occurrence, including which water bodies may bloom under specific weather conditions, affording local public health officials and resource managers the opportunity to forecast events and issue warnings before conditions become dangerous.

**Table 3:** Numbers of suspected or confirmed cyanoHAB-associated canine poisonings in the U.S., and estimated numbers of dog illnesses or deaths captured by three datasets: Harmful Algal Bloom-related Illness Surveillance System (HABISS), Media search, and Veterinary Medical Teaching Hospital (VMTH) records.

Number reported	Source of reports Dates of reports		
	HABISS 2007–2011	Media Search Late 1920s–2012	VMTH 1984–2011
Number of discrete cyanoHAB events associated with canine illness or death reported during the monitoring period	55	115	44
Number of sick or dead dogs attributed to cyanotoxin exposure across all reported cyanoHAB events during the monitoring period	63	260	45
Number of sick or dead dogs attributed to anatoxin-a or anatoxin-a(s) exposure	12	44	2
Number of sick or dead dogs attributed to microcystin or other hepatotoxic cyanotoxin exposure	3	5	43
Number (%) of cases where cyanoHAB intoxication was biochemically confirmed	8 (13%)	20 (8%)	2 (4%)
Number (%) of cases that were published in peer-reviewed scientific literature	0	62 (25%)	1 (2%)

In contrast to the media reports and published scientific manuscripts on cyanoHAB events that were summarized in the media database, HABISS was created to identify long-term trends for diverse marine and freshwater HAB events in the U.S., to assess the extent and severity of associated animal illness, and to clarify the nature of public health threats from these events. The objectives were to expand upon a pre-existing public health surveillance framework to facilitate collection of medical information from people and animals, and to include environmental data describing HAB event characteristics over time. Collected information would provide an historical dataset for examining temporal trends for cyanoHAB events and identifying human and animal risk factors for exposure.

Our retrospective review of canine accessions from a large veterinary teaching hospital in California provides a unique perspective on the number of suspected or confirmed cyanotoxin cases in dogs that can be identified through retrospective necropsy or biopsy accessions. We found that the proportion of cases that received biochemical testing was low, and the proportion of cases that were reported in the scientific literature was even lower. These data illustrate that the vast majority of cyanoHAB-associated dog deaths remain un-reported and often un-recognized by pet owners and veterinarians. Cyano-HAB-exposed animals do not receive specific treatment and communities may miss potential health hazards for other animals and humans. Our preliminary data suggest that the average age of microcystin-poisoned dogs may be older, and the average duration of illness longer than for dogs presenting with mushroom poisoning; however, additional investigation is needed. Specific testing and review of hospital case records from other geographic areas may provide additional insight on the age range of affected dogs, the onset and duration of illness and other factors that could help veterinarians to distinguish between similar disease syndromes, such as microcystin intoxication and mushroom poisoning.

These three datasets represent a snapshot of the range of cyanoHAB events occurring across the U.S. Education of the general public and medical personnel, more cost-effective and broadly available diagnostic tests, and surveillance efforts might improve our understanding of the risks to animals and humans from cyanoHAB exposure.

In the current study, all but one of the reports of dog illness and death were associated with freshwater cyanoHAB exposure. Toxic cyanobacterial blooms develop in diverse fresh and brackish water sources, including drainage ditches, culverts, ponds, lakes, reservoirs, rivers, streams and estuaries, especially those characterized by abundant sunlight; elevated nitrogen and/or phosphorus loads; and shallow, warm, still water. Once formed, cyanotoxins are relatively stable in water, surface scums, crusts and sediment, and can bioconcentrate in local biota, such as bivalves, shrimp, crabs, and fish [181,182,183,184]. Benthic cyanobacteria mats are [185] another potential source of toxicity, especially in rivers. Toxins and toxin-containing cyanobacteria can also flow downstream and contaminate estuarine and marine habitats, posing additional risks for domestic animals, humans and wildlife [184,186].

Domestic animals and wildlife can serve as sentinels for potential human health risks from a number of environmental pollutants, including pesticides and asbestos [187]. Pet dogs may be especially valuable sentinels for environmental contaminant exposure because they commonly live in close proximity with their owners and share similar lifestyles. For HABs, dogs are particularly valuable because of a particular component of their “lifestyle”—they will swim in, or drink from a scummy, smelly water body that people would avoid. In many cases, reports of acute dog illness or death after swimming provides the first warning that a toxin-producing bloom is present in a local water body. Because these toxins can aerosolize and can persist after a bloom has dissipated, and can exert subacute and chronic effects on humans, detection of cyanotoxicosis in dogs could alert local and national public health authorities to potential human health risks. Alerts regarding the increased frequency and broad distribution of toxic cyanobacterial blooms throughout the U.S. could help veterinarians identify “high-risk” sources of exposure. Further, an improved understanding of symptoms, clinical progression and pathophysiology of cyanotoxin poisonings could help animal health professionals to more accurately and quickly diagnose HAB-related illness and facilitate treatment. One option to achieve that understanding might be for veterinarians to share general case information with local physicians to both alert them to the possibility of human exposure and encourage them to ask about potential water or cyanobacterial contact when patients present with relevant symptoms [12].

HAB events will likely become more common and severe over time [4]. Global climate change, increased water withdrawal, lower water tables and extended drought can enhance algal blooms by decreasing water flow, concentrating nutrients and increasing water temperatures. Exponential increases in fertilizer used globally have resulted in nutrient loading of riparian systems, further enhancing cyanobacterial bloom development and toxin production [188,189]. Precipitation washes nutrients into water bodies and may transport blooms and cyanotoxins downstream or into new areas. For example, Miller *et al*. [184] reported poisoning of 21 sea otters by microcystins transported from local rivers and streams to the ocean in the Monterey Bay region of California. Interestingly, between 2007 and 2010, at least 8 dogs developed clinically significant or fatal liver disease after visiting Monterey-area beaches (Miller, unpub. data). Although 2 of these dogs belonged to local veterinarians, no diagnosis was made and no microcystin testing was done. The veterinary and human medical communities, public health officials, and natural resource managers could be included in education and outreach activities to improve recognition and appropriate diagnosis of HAB-related exposures and health outcomes. NCEH and many states and have created HAB-related materials, including brochures, tools to create HAB response plans, signs, and other communication materials. Table 1 lists a few of the many websites where HAB-related information is available.

Active surveillance for HAB-related illnesses in people and animals could increase our knowledge about the occurrence and distribution of these illnesses. Novel approaches to address HAB issues include citizen participation to facilitate data collection and close knowledge gaps. One example is the Phytoplankton Monitoring Network (PMN) supported by the National Oceanographic and Atmospheric Administration (NOAA) [190], which provided hands-on training and basic equipment for community members to monitor local inland water bodies or nearby ocean shores for algal blooms. NOAA scientists screen the results and maintain it in a database accessible to volunteers. Another novel approach is use of neighborhood networking programs to share local environmental information such as NeighborHound Watch, where pet owners can post details about toxic algal blooms to alert other pet owners of potential hazards in their local area [191].

## 3. Experimental Section

We examined a range of retrospective information sources for possible or confirmed cases of dog poisoning due to cyanotoxin exposure to better estimate the extent of canine morbidity and mortality resulting from exposure to freshwater cyanoHABs in the United States (U.S.). Case information was obtained from the following three sources: (1) The Harmful Algal Bloom-related Illness Surveillance System (HABISS) of the National Center for Environmental Health (NCEH), Centers for Disease Control and Prevention (CDC); (2) Retrospective case files from a large, regional veterinary hospital (Veterinary Medical Teaching Hospital [VMTH], University of California, Davis); and (3) Publicly available scientific and medical manuscripts; written media; and web-based reports from pet owners, veterinarians, or other individuals throughout the U.S. Data in HABISS represents systematic surveillance for HAB-related diseases from 2007–2012, and the survey of publically available data spanned accessible reports from the 1920s–2012. We examined cases from each dataset individually and as pooled data.

### 3.1. Harmful Algal Bloom-Related Illness Surveillance System (HABISS)

In response to the need to support public health decision-making about health risks associated with exposure to HABs and associated toxins, NCEH developed the HABISS, which closed for data collection in 2012 [192]. HABISS was a unique surveillance system designed to capture human and animal health data as well as physical characteristics of HABs in a single database. HABISS was an active surveillance system operating on NCEH’s secure platform—the Rapid Data Collector (RDC)—a tool designed in-house specifically for this effort. Protected by approved access certificates and passwords, state users could enter, edit, and save data for subsequent sessions. HABISS required users to input several key indicators to expedite data retrieval, including dates of bloom, agency contact information, state codes, route of exposure, and patient complaints. If additional data were available, users could report data elements for suspected human or animal illness, including point of contact with HAB, demographics, symptoms, test results, and interim and final diagnoses. The system linked human illness reports and animal illness and mortality reports and data collected on relevant blooms. Key elements for the bloom report included water body name and location, water sample collection methods, analytic testing results, algal counts, and algal taxonomy. Data were exportable to Access, Excel, or XML for analysis.

Case definitions for HAB-associated animal illnesses and deaths in HABISS were as follows: A suspect case included exposure to water with a confirmed algal bloom, onset of associated signs and symptoms within a reasonable time after exposure, and no other cause of illness. A probable case met criteria for suspect case and includes laboratory-based documentation of a HAB-related toxin in the water. A confirmed case met criteria for probable case combined with professional judgment based on medical review, or, met criteria for probable case with documentation of a HAB-related toxin in a clinical specimen.

NCEH collected reports of human and animal illnesses associated with exposure to HABs from 2009 until September 2012 from 13 states using HABISS. Reports were received primarily from the 9 states funded by NCEH’s Cooperative Agreement to Enhance Surveillance of Risk Factors and Health; however, other states also contributed data. Pet owners whose dogs became ill or died following exposure to HABs and veterinarians who treated exposed dogs provided information to the states or to NCEH. States entered data from historic events and from events that occurred through the calendar year 2011. This summary focuses on events reported to HABISS that occurred between January 2007 and 8 July 2011.

### 3.2. Potential and Confirmed Canine CyanoHAB Cases from the Veterinary Medical Teaching Hospital (VMTH), University of California, Davis

The Veterinary Medical Teaching Hospital (VMTH) at UC, Davis is a regional veterinary hospital with a catchment area in Northern and Central California. Retrospective VMTH biopsy and necropsy case files for canine accessions were searched between 1984 and 2012 for keywords encompassing the subject-areas of acute hepatic failure, hepatotoxin, blue-green algae, cyanobacteria, cyanotoxin, mushroom, amatoxin, amanitin, microcystin and anatoxin. A veterinary pathologist who was familiar with cyanotoxin-associated pathology reviewed all available historical and necropsy data for each case matching these keywords, and categorized each dog according to its relative likelihood of being a cyanotoxin poisoning case (low, moderate or high). We noted the implicated cyanotoxin (e.g., hepatotoxin, such as microcystin, or neurotoxin, such as anatoxin) for any cases of possible or confirmed cyanotoxicosis. We abstracted the following information for each case: animal location, pathology number, patient number, breed, age, sex, whether or not there was an associated algal bloom, location of exposure (if known), county of exposure, duration of illness, date of death or biopsy request, whether the animal was euthanized, tissue condition, nutritional status, clinical history, histopathology results, microbiological results, and case summaries.

The retrospective review of case files from the VMTH provided a unique perspective on the proportion of dogs presenting with compatible clinical signs and pathology that was tested for cyanotoxins, and the proportion of cases where cyanotoxin poisoning was a differential diagnosis. Differential diagnoses for acute hepatic failure in dogs include poisoning by cyanotoxins like microcystin, nodularin or cylindrospermopsin, cyanotoxins associated with consumption of cycad palms, toxins from poison mushrooms such as amanitin, fungal toxins (e.g., aflatoxin); pharmaceuticals (e.g., acetaminophen and carprofen, or Rimadyl), anticoagulant pesticides, metals (e.g., iron and copper), phosphorous, phenolic compounds, coal tar, and xylitol. Microcystin appears to be a common cyanotoxin affecting dogs in the U.S. [18,24,95,168] and was one focus of the current study. Because identifying causes of acute hepatic failure can be difficult and costly, few cases had confirmatory tests performed for multiple hepatotoxins. Thus, each case was categorized by the most likely cause of the observed lesions (cyanotoxin intoxication, mushroom poisoning, or other hepatotoxin) based on the clinical history, lesion description, necropsy/biopsy results, case summary, and results of any available toxicological tests. Many factors resulted in categorization as a higher likelihood of microcystin poisoning, including: (1) Cyanobacteria or microcystin detection in animal, water or algal scum sample; (2) History of exposure to a bloom or cyanotoxin-contaminated water, or (3) History of swimming. We also categorized a case as a higher likelihood of microcystin poisoning if cyanotoxin poisoning was included as a differential diagnosis in the pathology report or if we found histological confirmation of hepatic lesions consistent with microcystin exposure (*i.e.*, mid-zonal, centrilobular or panlobular hepatocellular apoptosis, necrosis, marked cell swelling and cytoplasmic vacuolation, parenchymal hemorrhage, hepatocyte dissociation, enlarged or atypical nuclei or frequent binucleate cells). Finally, a case was categorized as a higher likelihood of microcystin exposure if there was no known or confirmed exposure to other acute hepatotoxins or neurotoxins (e.g., amanitin or anti-inflammatory medications) or if we found scant prior history of mushroom consumption.

### 3.3. Historical Reports from Scientific Publications, Media and Electronic Sources

We conducted a retrospective review for all cases of cyanoHAB-related dog illnesses and deaths in the U.S. reported in peer-reviewed scientific, veterinary, and medical literature, gray literature, and scientific proceedings using PubMed, a medical abstracting electronic database. We also reviewed media reports, including newspaper archives, internet resources identified using the Google^©^ search engine, and anecdotal data collected through personal communications with dog owners and veterinarians. We did not specify a time-frame. We collected 115 reports of possible or confirmed canine cyanotoxin poisonings. Data abstracted or derived from the reports included (when available) date of report, date of exposure, type of water body (pond, reservoir, lake, or river), location of exposure (county, state), cyanoHAB species if identified, toxin if identified, number of dogs affected, dog breed(s), clinical signs and observations, test results and diagnoses, and reference for the report. 

### 3.4. Overlap of Canine Cyanotoxin Intoxication Cases Identified Using the Three Sources

We reviewed each report describing a cyanoHAB-related canine illness or death in the three datasets to identify duplicates where possible. For comparison purposes, all results were restricted to exposures that occurred between January 2007 and July 2011 to optimize comparability with HABISS. We matched case reports using the state where the event occurred, the month and year of exposure, and the associated water body, if known. We also used additional information, such as dog breed, when available. Of the 46 case reports captured through the media search, 11 (24%) were also captured in HABISS. Of these 11 events (16 cases), 8 were entered into HABISS by states funded by NCEH. The other three events were identified by NCEH using a daily Google^©^ search for media reports on cyanobacteria/blue green algae. We then contacted the states where the events occurred, CA and KS, to follow up and obtain data for HABISS. Two additional events captured by the media review were very similar to events reported in HABISS, but the number of affected dogs was different. For example, a media report from 14 June 2007 noted the death of a yellow Labrador retriever following swimming in Fountain Lake, MN. HABISS contained a single event involving three dogs in MN during that month. Of 11 dogs in the VMTH dataset that presented during the 2007–2011 timeframe, none was duplicated in HABISS. There were also no overlaps between the data from the media search and the VMTH dataset. 

### 3.5. Limitations

The primary limitation for this review of cases of canine cyanotoxin poisonings was the limited catchment area for reports collected using HABISS (limited to 13 states) and the VMTH dataset (limited to California). Another limitation was that we could not verify exposure in cases reported before analytic methods were developed to identify and quantify cyanotoxins in water or animal tissues. Finally, HABISS was passive surveillance; thus, states were encouraged, but not required, to report cases, and so the actual number of cases was likely higher than reported. 

## 4. Conclusions

The acute canine cyanotoxin poisonings reviewed here likely represent only a small fraction of cases that occurred throughout the U.S. during the time periods represented by three different datasets. This paper demonstrates that a number of factors, including a lack of veterinary training to recognize cyanotoxin poisoning; limited access to validated, cost effective diagnostic tests; and a tendency to not publish cases when encountered by veterinarians, collectively are likely resulting in significant under-recognition, and thus under-reporting, of these important health events. Evidence and experience suggest there are a number of options with potential to increase recognition and reporting of, and response to, HAB-related health events. These options include: (1) creating national maps of historical and current cyanoHAB events; (2) obtaining more information about exposure and clinical aspects of these poisonings so they can be quickly and accurately diagnosed and treated; (3) disseminating outreach materials to the medical and veterinary medical communities; (4) developing cost-effective diagnostic methods that are more widely available; (5) publishing case reports to document cyanoHAB poisonings and supporting rapid dissemination of results of successful treatment and animal care; (6) enhancing integration and communication between natural resource managers, the public, and the veterinary and public health community about cyanobacteria risks to pets; (7) increasing monitoring and identification of toxic cyanobacteria in managed water bodies and those used for recreation; and (8) posting more signs and warnings to the public about the threat to pets in high risk areas.

## References

[1] Sivonen K., Jones G., Chorus I., Bartram J. (1999). Cyanobacterial Toxins. Toxic Cyanobacteria in Water. A Guide to Their Public Health Consequences, Monitoring and Management.

[2] Stewart I., Carmichael W., Backer L., Selendy J. (2011). Cyanobacteria. Water and Sanitation-Related Diseases and the Environment: Challenges, Interventions and Preventive Measures.

[3] Backer L., McGillicuddy D.J. (2006). Harmful algal blooms at the interface between coastal oceanography and human health. Oceanography.

[4] Backer L., Moore S., Nemr A. (2010). Harmful Algal Blooms: Future Threats in a Warmer World. Environmental Pollution and Its Relation to Climate Change.

[5] Paerl H.W., Paul V.J. (2012). Climate change: Links to global expansion of harmful cyanobacteria. Water Res..

[6] Stewart I., Webb P.M., Schluter P.J., Fleming L.E., Burns J.W., Gantar M., Backer L.C., Shaw G.R. (2006). Epidemiology of recreational exposure to freshwater cyanobacteria—An international prospective cohort study. BMC Public Health.

[7] Backer L.C., Carmichael W., Kirkpatrick B., Williams C., Irvin M., Zhou Y., Johnson T.B., Nierenberg K., Hill V., Kieszak S.M. (2008). Recreational exposure to low concentrations of microcystins during an algal bloom in a small lake. Mar. Drugs.

[8] Backer L.C., McNeel S.V., Barber T., Kirkpatrick B., Williams C., Irvin M., Zhou Y., Johnson T.B., Nierenberg K., Aubel M. (2010). Recreational exposure to microcystins during algal blooms in two California lakes. Toxicon.

[9] Backer L.C. (2002). Cyanobacterial harmful algal blooms (CyanoHABs): Developing a public health response. Lake Reserv. Manag..

[10] Senior V.E. (1960). Algal poisoning in Saskatchewan. Can. J. Comp. Med. Vet. Sci..

[11] Codd G.A., Edwards C., Beattle K.A., Barr W.M., Gunn G.J. (1992). Fatal attraction to cyanobacteria?. Nature.

[12] Zaias J., Backer L., Fleming L., Rabinowitz P., Conti L. (2010). Harmful Algal Blooms. Human-Animal Medicine Clinical Approaches to Zoonoses, Toxicants, and Other Shared Health Risks.

[13] Landsberg J.H. (2002). The effects of harmful algal blooms on aquatic organisms. Rev. Fish. Sci..

[14] Beasley V.R., Cook W.O., Dahlem A.M., Hooser S.B., Lovell R.A., Valentine W.M. (1989). Algae intoxication in livestock and waterfowl. Vet. Clin. North Am. Food Anim. Pract..

[15] Beasley V.R., Dahlem A.M., Cook W.O., Valentine W.M., Lovell R.A., Hooser S.B., Harada K., Suzuki M., Carmichael W.W. (1989). Diagnostic and clinically important aspects of cyanobacterial (blue-green algae) toxicoses. J. Vet. Diagn. Investig..

[16] Cheng Y.S., Zhou Y., Irvin C.M., Kirkpatrick B., Backer L.C. (2007). Characterization of aerosols containing microcystin. Mar. Drugs.

[17] Codd G.A., Bell S.G., Kaya K., Ward C.J., Beattie K.A., Metcalf J.S. (1999). Cyanobacterial toxins, exposure routes and human health. Eur. J. Phycol..

[18] Rankin K., Alroy K., Kudela R., Oates S., Murray M., Miller M.A. (2013). Treatment of cyanobacterial (microcystin) toxicosis using oral cholestyramine: Case report of a dog from Montana. Toxins.

[19] Mereish K.A., Solow R. (1990). Effect of antihepatotoxic agents against microcystin-LR toxicity in cultured rat hepatocytes. Pharm. Res..

[20] Mereish K.A., Bunner D.L., Ragland D.R., Creasia D.A. (1991). Protection against microcystin-LR-induced hepatotoxicity by Silymarin: Biochemistry, histopathology, and lethality. Pharm. Res..

[21] Rao P.V.L., Jayaraj R., Bhaskar A.S. (2004). Protective efficacy and the recovery profile of certain chemoprotectants against lethal poisoning by microcystin-LR in mice. Toxicon.

[22] Rao P.V.L., Gupta N., Jayaraj R. (2004). Screening of certain chemoprotectants against cyclic peptide toxin microcystin-LR. Indian J. Pharmacol..

[23] Kurek J. (2013). Managing the Acutely Convulsing Patient: Generalized Tremors, Cluster Seizures, and Status Epileptics. Pacific Tide.

[24] DeVries S.E., Galey F.D., Namikoshi M., Woo J.C. (1993). Clinical and pathologic findings of blue-green algae (*Microcystis aeruginosa*) intoxication in a dog. J. Vet. Diagn. Investig..

[25] California Department of Water Resources (1966). Clear Lake Water Quality Investigation.

[26] Schwimmer D., Schwimmer M., Jackson D. (1968). Medical Aspects of Phycology. Algae, Man and the Environment.

[27] (1944). Pigs Die after Drinking Water from OKOBOJI. Vindicator and Republican.

[28] Rose E.T. (1953). Toxic algae in Iowa lakes. Proc. Iowa Acad. Sci..

[29] Yoo R.S., Carmichael W.W., Hoehn R.C., Hrudey S.E. (1995). Cyanobacterial (Blue-Green algal) Toxins: A Resource Guide.

[30] (1952). Lake Water not Only Made Dog Sick—It Killed Animal!. LeMars Globe Post.

[31] Firkins G.S. (1953). Toxic algae poisoning. Iowa State Coll. Vet..

[32] Brown L. (1973). Report Nearly Completed on Dying Lake. The Lakeland Ledger.

[33] (1976). Lake Kills Only Dogs. The Spokesman Review.

[34] Young L. (1978). Toxic Algae Discovered in Spokane Arm of Lake Roosevelt. The Spokesman Review.

[35] Soltero R.A., Nichols D.G., Carmichael W.W. (1981). The Recent Blue-Green Algal Blooms of Long Lake, Washington. The Water Environment: Algal Toxins and Health.

[36] Jacoby J.M., Kann J. (2007). The occurrence and response to toxic cyanobacteria in the Pacific Northwest, North America. Lake Reserv. Manag..

[37] (1977). Forest Service Officials Eye Hebgen Lake Closure. Idaho Falls Post Register.

[38] (1977). Hebgen Lake: Swimming Barred After 14 Cows Die. The Spokesman Review.

[39] Juday R.E., Keller E.J., Horpestad A., Bahls L.L., Glasser S., Carmichael W.W. (1981). A toxic bloom of *Anabaena Flos-Aquae* in Hebgen Reservoir Montana in 1977. The Water Environment: Algal Toxins and Health.

[40] (1977). Warning Signs Planned. Spokane Daily Chronicle.

[41] Billings W.H., Carmichael W.W. (1981). Water-Associated Human Illness in North-East Pennsylvania and Its Suspected Association with Blue-Green Algal Blooms. The Water Environment: Algal Toxins and Health.

[42] (1980). Toxic Algae Blooming. Spokesman Review.

[43] Associated Press (1982). Algae Fouls Lake, Kills Dogs, Cows. Spokane Chronicle.

[44] Associated Press (1982). Dogs, Cows Die after Drinking Polluted Water. Lewiston Morning Tribune.

[45] Kann J., Falter C.M. (1985). Blue-Green Algae Toxicity in Black Lake, Kootenai County, Idaho.

[46] (1985). Access to 2 Lakes Restricted after the Deaths of 2 Dogs. The Milwaukeee Journal.

[47] Sonzogni W.C., Repavich W.M., Standridge J.H., Wedepohl R.E., Vennie J.G. (1988). A note on algal toxins in Wisconsin waters experiencing blue-green algae blooms. Lake Reserv. Manag..

[48] Mahmood N.A., Carmichael W.W., Pfahler D. (1988). Anticholinesterase poisonings in dogs from a cyanobacterial (blue-green algae) bloom dominated by *Anabaena flos-aquae*. Am. J. Vet. Res..

[49] Dadisman Q. (1987). Toxic Algae Linked to Death of Cows, Dogs. The Milwaukee Sentinel.

[50] (1986). Algae Prompting Clifton to Empty Main Memorial Pond. The Record.

[51] (1989). Demise of Herons Laid to Algae. Star Tribune.

[52] Associated Press (1989). Health Officials Blame Cats’ Deaths on Algae Toxin in Lake. Anchorage Daily News.

[53] Associated Press (1989). Algae Bloom in Lake Baffles Experts. Eugene Register Guard.

[54] Associated Press (1989). Eight Pets Killed by Poisonous Algae Bloom. Spokane Chronicle.

[55] Winterton D. (2011). Report: Toxic Algae in East Canyon Reservoir. Standard Examiner.

[56] Environmental Protection Agency (EPA) (2010). Lake Washington—No Longer Muddying Up the Waters. Section 319 Success Stories.

[57] (1990). Algae Threat Gone from Lake. The Commercial Appeal.

[58] Carmichael W.W. (1991). Toxic freshwater blue-green algae (cyanobacteria): An overlooked health threat. Health Environ. Digest..

[59] (1991). Deadly Blue-Green Algae was Found in Mina Lake. USA Today.

[60] (1991). Algae Kills Dogs. Ellensburg Daily Record.

[61] (1991). Five Hunting Dogs Killed by Algae in Drinking Water. The Oregonian.

[62] McAllister T. (1991). Hunters Share Sad Story of Dogs Poisoned by Algae to Alert Others to Danger. The Oregonian.

[63] Gorham M.E. (1997). Blue-Green Algae can be Deadly to Dogs, Other Animals. The Columbian.

[64] Marks J. (1997). Water in California’s Carmel River is Deemed Unsafe to Drink. Monterey County Herald.

[65] Calvan B.C. (1997). Is Dog Toxic Lake’s First Victim?—Officials Concerned over Spreading Microbes. The Seattle Times.

[66] Johnston B.R., Jacoby J.M. (2003). Cyanobacterial toxicity and migration in a mesotrophic lake in western Washington, USA. Hydrobiologia.

[67] (1998). Algae Prompts DNR to Issue Lake Warning. St. Paul Pioneer Press.

[68] (1998). Maryland Department of Agriculture: Protect Animals from Blue-Green Algae. PR Newswire.

[69] Leaning J. (1998). Toxic Algae Killed Dogs at Nickerson. Cape Cod Times.

[70] Leaning J. (1998). Fourth Dog Sickens in Park. Cape Cod Times.

[71] Moore S., Lanphear M. (1999). Dog Deaths Laid to Poison Lake Algea. Press Republican.

[72] Moore S. (1999). Lake Algae Blooms Still Pose Threat. Press Republican.

[73] Boyer G., Watzin M.C., Shambaugh A.D., Satchwell M.F., Rosen B.R., Mihuc T., Manley T.O., Manley P.L., Mihuc T.B. (2004). The Occurrence of Cyanobacterial Toxins in Lake Champlain. Lake Champlain: Partnerships and Research in the New Millennium.

[74] Boyer G.L. (2008). Cyanobacterial toxins in New York and the lower Great Lakes ecosystems. Adv. Exp. Med. Biol..

[75] Bazilchuk N. (1999). Toxin in Lake Champlain Algae Kills Two N.Y. Dogs. The Burlington Free Press.

[76] (1999). Threat of Troubled Waters. Dog Deaths Lead Officials to Check River. Times News.

[77] (1999). Mystery of Dog Death Deepens. Times News.

[78] Associated Press (2000). Scientists: Dog Died Because of Algae. Lewiston Morning Tribune.

[79] (2000). Scientists Confirm Algae Killed Dog Near Lake Lowell. Times News.

[80] (2000). Officials Wait on Final Report of Dog Deaths. USDA to Release Preliminary Findings on Algae Next Week. Times News.

[81] (2000). Second Dog Dies Along Snake. Times News.

[82] (2000). Toxicologists Confirm Snake River Algae Species. The Times-News.

[83] Hill H. (2005). Dog Deaths in Humboldt and Mendocino County Water Bodies Possibly Related to Cyanobacterial Toxicity. http://www.waterboards.ca.gov/water_issues/programs/bluegreen_algae/docs/workgroup110805/harriethill.pdf.

[84] Holschuh A. (2001). Danger on the Beach. Is Toxic Algae Killing Dogs at Big Lagoon?. North Coast Journal Weekly.

[85] Soussan T. (2002). Warning Issued for Dogs at Lake. Albuquerque Journal.

[86] Thilsted J. (2002). New Mexico Department of Agriculture, Veterinary Diagnostic Services, Elephant Butte canine poisoning cyanobacteria. Personal communication.

[87] Puschner B., Hoff B., Tor E.R. (2008). Diagnosis of anatoxin-a poisoning in dogs from North America. J. Vet. Diagn. Investig..

[88] Associated Press (2002). Algae Warning Issued by State. Times Argus.

[89] Silverman A. (2002). Algae Suspected in Dog Deaths. The Burlington Free Press.

[90] Ansami R. (2003). Deadly Algae Invades Lakes. Daily Globe.

[91] Smith D. (2003). Algae Blamed for Dogs’ Death. Star Tribune.

[92] Gaarder N. (2004). Toxin Closes Buccaneer Bay’s Lake. Omaha World Herald.

[93] Laukaitis A.J. (2004). Toxic Algae Closes Buccaneer Bay Lake. Lincoln Journal Star.

[94] Brakhage P.A. (2009). Cyanobacteria, the Nebraska Experience.

[95] Walker S.R., Lund J.C., Schumacher D.G., Brakhage P.A., McManus B.C., Miller J.D., Augustine M.M., Carney J.J., Holland R.S., Hoagland K.D. (2008). Nebraska experience. Adv. Exp. Med. Biol..

[96] (2004). Sarpy Lakes Closed. Channel 6 News.

[97] Gaarder N. (2004). Lake Algae Blamed for Dog Deaths. Water trouble. Omaha World Herald.

[98] Goldberg D. (2004). Lake Algae may be Killing Animals, Birds. Syracuse Post Standard.

[99] Goldberg D. (2004). Advisories Posted at Lake; Toxic Algae Prompts Officials to Close Lake Neatahwanta. Syracuse Post Standard.

[100] Spencer K.M., Boyer G. (2005). Dog Deaths of Summer on Lake Neatahwanta. Upper Peninsula Student Research Symposium in Chemistry and Related Fields.

[101] Sensenbrenner L. (2004). Pond Algae is Blamed for Pet Dog’s Seizures. Capital Times.

[102] Novak B., Sensenbrenner L. (2004). Toxin Scare Hits Area Lakes; Kegonsa Lake Closed after Dog Suffers Convulsions. The Capital Times.

[103] Williams B. (2004). Dog Dies after Swim in Kegonsa. Algae Toxin in Water may be to Blame; Officials will Still Test. Wisconsin State Journal.

[104] Lindon M., Heiskary S. (2007). Microcystin Levels in Eutrophic South Central Minnesota Lakes.

[105] Jacoby J. (2004). Brownless Algae may be Deadly to Dogs. Baker City Herald.

[106] Fode M. (2004). Blue-Green Algae Bloom Causes at Least One Dog Death at Lake Benton. Pipestone County Star.

[107] Leventis A. (2004). Toxic Algae Claims Another Family Pet; Blue-Green Algae in Steilacoom Lake Likely Killed a Family Dog. Lake Homeowners are Running out of Options to Treat the Toxic Algae. The News Tribune.

[108] Hanowell R. (2005). Toxic Blue-Green Algae Blooms. Tacoma Pierce County Health Department Veterinary Newsletter.

[109] Novak B. (2005). Toxic Algae Warning Issued; Scum on Area Lakes, Ponds Threaten People, Pets. The Capital Times.

[110] (2005). Popular Pond may be Contaminated. The Hawk-Eye.

[111] (2006). Toxic Algae on Private Lakes. WOWT.com News.

[112] (2006). Toxic Algae Surfaces at Thomas Lakes. Suburban Newspapers.

[113] McCormick J. (2006). Toxic Algae may Have Killed 2 Dogs at Lake. Kitsap Sun.

[114] Hardy J. (2008). Dog Deaths from Cyanobacteria Spurs Lake Monitoring. Zoonotic Disease Newsletter.

[115] Zimowsky P. (2006). Algae in Some Idaho Waters can Sicken or Kill Dogs. The Idaho Statesman.

[116] Debner M. (2007). Toxic Algae Found in Lakes Linked to dog’s Death. Angler Insider.

[117] Marohn K. (2007). Dog’s Death Spurs Algae Warning. St. Cloud Times.

[118] Smith D. (2007). Outdoors Almanac: Four Dogs Have Died from Blue-Green Algae Poisoning. Star Tribune.

[119] Machniak C. (2007). ‘The Water in This Lake Killed My Dog’; Dog Owner, Neighbors at Odds over Canine’s Cause of Death. Flint Journal.

[120] (2007). Lake Algae is Focus in Probe of Dog’s Death. Detroit Free Press.

[121] Smith D. (2007). Blue-Green Algae Poisoning Has Killed Four Dogs. Star Tribune.

[122] Ratzlaff A. (2007). Dog Deaths at Local Reservoir may be Linked to Algae Toxins. Hillsboro Free Press.

[123] (2007). Algae Blooms Forming on State Waters Again. KULR8.com.

[124] Hamel K. (2008). Watch for Cyanobacteria “Blue-Green Algae” on Your Lake. Waterline.

[125] Kiava L. (2007). 2 More Minn. Dogs Dead from Blue-Green Algae. WCCO TV.

[126] (2007). Caution Urged after Dog Dies at Elephant Butte. Las Cruces Sun-News.

[127] (2007). Elephant Butte Algae may Have Killed Dog. El Paso Times.

[128] (2007). Possible Algae Poisoning at Elephant Butte. Ruidoso News.

[129] Cardin F. (2007). We must Address the Problem of Blue-Green Algae. The Post-Crescent.

[130] McCartney D. (2008). Danger to Dogs can Lurk in Pond Water, Woman Finds. The Wichita Eagle.

[131] Chase C. (2010). Toxic Algae Bloom Poisons Dog. Daily Interlake.com.

[132] (2008). DNR Cautions Dog Owners about Fatal Algae Toxin. The Fulton County News.

[133] (2009). Stinky Blue-Green Algae Blamed for Dog Deaths.Algae is Blooming in Response to Drought and Fertilizer Runoffs from Farms. NBCnews.com.

[134] Goiffon T. (2009). Dog Deaths and Blue Green Algae. Aussie Talk.

[135] Hamel K. (2009). Freshwater Algae Control Program: Report to the Washington State Legislature (2008–2009).

[136] Meeks A. (2009). Algae in Lake Water Suspected in Dogs’ Deaths. Alamogordo Daily News.

[137] Meeks A. (2009). Algae in Elephant Butte Water Suspected in Dogs’ Deaths. Las Cruces Sun-News.

[138] North Dakota State University Extension Service (2009). It’s not Too Early to Watch for Blue-Green Algae. Agweek.

[139] Burns R. (2009). Killer Algae on the Van Duzen. The Journal.

[140] (2009). California Regional Water Quality Control Board, Agencies Warn to Avoid Exposure to Toxic Blue Green Algae in Three Northern CA Rivers. News Release.

[141] Bolt G. (2009). Tests Find Dog Died of Toxin in Algae. All Four Dogs that were at Elk Creek Near Elkton Now are Presumed to Have Died from the Water. The Register Guard.

[142] Terry L. (2009). OSU Confirms Algae Killed Dogs. The Oregonian.

[143] Rillos L. (2009). Four Dogs Die: ‘Something Seriously Toxic is on the Banks’. KVAL.COM.

[144] Freeman M. (2009). Dog Dies from Toxic Algae, Lab Tests Reveal. Health Officials Hope This will be a Wake-Up Call for People to Stay Away from Water When Warned. Mail Tribune.

[145] Lyon C. (2011). DWP Develops resources for cyanobacteria and drinking water. Pipeline.

[146] (2009). Dogs are Endangered by Lake Algae. Antigo Daily Journal.

[147] (2009). River: Slow Flow Merits Caution. San Marcos Daily Record.

[148] Lien D. (2009). Algae Exposure Suspected in Death of Dog in Southern Minnesota. Pioneer Press.

[149] (2009). Dog Dead from Fox Lake Algae. Jackson County Pilot.

[150] Associated Press (2009). Dog dies after exposure to toxic algae in Twin Cities. Post Bulletin.com.

[151] Imrie R. (2009). What’s Ugly, Smells, Kills Dogs? Blue-Green Algae. Seattle Times.

[152] Robinson E. (2009). Lacamas, Round Lakes Closed after Pet Dog Dies. Columbian.

[153] Lowe S. (2009). Warning Dog Owners: Blue-Green Algae can be Possible Hazard. South Bend Tribune.

[154] Polk County Association of Lakes and Rivers (2010). Meeting Minutes. http://pcalr.org/wppcalr/wp-content/uploads/2011/12/Minutes_102010.pdf.

[155] (2010). Dog Dies, Owner Ill after Animal Swims in Algae-Choked Grand Lake St. Marys. Sandusky Register.

[156] Hunt S., McGlade C. (2010). Algae may be Killing Pets; At Least Three Dogs Dead, Nine Humans Ill. The Columbus Dispatch.

[157] (2010). Low Toxin Levels Found in Burr Oak State Park Lake. The Athens News.

[158] North Dakota Department of Health (2010). State Health Department Issues Blue-Green Algae Bloom Advisory. http://www.ndhan.gov/data/mrNews/Harmful%20Algae%20Lake%20Josephine.pdf.

[159] Freeman M. (2010). Algae Toxin Levels Prompt Alarm. Blue-Green Algae Registers 400 Times Higher than Mark That Killed Dog. Mail Tribune.

[160] (2010). Algae Forces Closing of Honeoye Beach. Rochester Democrat and Chronicle.

[161] Lutz A. (2010). Flathead Dog Sick with Blue, Green Algae. KPAX.COM.

[162] Simpson N. (2011). Pembroke Pond Closed after Girls Get Sick. The Patriot Ledger.

[163] Freeman M. (2011). Health Warnings Issued after Algae Discoveries. Mail Tribune.

[164] Kansas State University (2011). Lethal Ingestion: Diagnostic Lab Analyzes Blue-Green Algae in Recent Dog Deaths. Lifelines.

[165] Creighton A. (2011). Yoncalla Dog Dies after River Outing, Blue-Green Algae Likely the Cause. The News Review Today.

[166] Ferenchik M. (2011). Toxic Algae in Pond could be Cause of Dog’s Death. The Columbus Dispatch.

[167] Ferenchik M. (2011). Tests Find no Algae Liver Toxin in Dog. The Columbus Dispatch.

[168] Van der Merwe D., Sebbag L., Nietfeld J.C., Aubel M.T., Foss A., Carney E. (2012). Investigation of a *Microcystis aeruginosa* cyanobacterial freshwater harmful algal bloom associated with acute microcystin toxicosis in a dog. J. Vet. Diagn. Invest..

[169] (2012). Blue-Green Algae is Believed to Have Killed to Dogs at Lake Ellsworth. 7 News KSWO.

[170] Buelow J. (2012). Blue-Green Algae Bloom Blamed for Dog’s Death. Tomahawk Leader.

[171] Widener A. (2012). Dogs Die after Swimming in Salamonie Reservoir. WANE.COM.

[172] Wiehe J. (2012). State Warns of Toxic Algae after Lake Trip Dooms Dogs. Fort Wayne Journal Gazette.

[173] Miller R. (2012). Toxic Blue-Green Algae Blooms in Area Waters. Olean Times Herald.

[174] (2012). Blue Green Algae. NeighborHound Watch.

[175] Cassoli D., Downes S. (2013). Cool cases from the E. R. Part 2. http://www.vcaspecialtyvets.com/ckfinder/userfiles/files/Northwest/Winterfest%202012/Cassoli%20and%20Downes%20-%20Cool%20Cases%20-%20Part%202.pdf.

[176] U.S. Environmental Protection Agency (2008). Reconsideration of California’s 2006 Section 303(d) List Omission of Microcystin Toxin Listings for Three Klamath River Segments and Determination to Add Microcystin Toxins Listing for Klamath River Hydrologic Unit (HU), Middle HA Hydrologic Area (HA), Oregon to Iron Gate.

[177] U.S. Environmental Protection Agency (2013). Climate Change Indicators in the United States. http://www.epa.gov/climatechange/science/indicators/weather-climate/temperature.html.

[178] Carmichael W.W., Evans W.R., Yin Q.Q., Bell P., Moczydlowski E. (1997). Evidence for paralytic shellfish poisons in the freshwater cyanobacterium *Lyngbya wollei* (Farlow ex. Gomont) comb. nov. Appl. Environ. Microbiol..

[179] Carmichael W.W., Biggs D.F., Gorham P.R. (1975). Toxicology and pharmacological action of *Anabaena flos-aquae* toxin. Science.

[180] Devlin J.P., Edwards O.E., Gorham P.R., Hunter N.R., Pike R.K., Stavric B. (1977). Anatoxin-a, a toxic alkaloid from *Anabaena flos-aquae* NRC-44h. Can. J. Chem..

[181] Negri A.P., Jones G.J. (1995). Bioaccumulation of paralytic shellfish poisoning (PSP) toxins from the cyanobacterium *Anabaena circinalis* by the freshwater mussel *Alathyria condola*. Toxicon.

[182] Kankaanpää H.T., Holliday J., Schröder H., Goddard T.J., von Fister R., Carmichael W.W. (2005). Cyanobacteria and prawn farming in northern New South Wales, Australia—A case study on cyanobacteria diversity and hepatotoxin bioaccumulation. Toxicol. Appl. Pharmacol..

[183] Smith J.L., Haney J.F. (2006). Foodweb transfer, accumulation, and depuration of microcystins, a cyanobacterial toxin, in pumpkinseed sunfish (*Lepomis gibbos*). Toxicon.

[184] Miller M.A., Kudela R.M., Mekebri A., Crane D., Oates S.C., Tinker M.T., Staedler M., Miller W.A., Toy-Choutka S., Dominik C. (2010). Evidence for a novel marine harmful algal bloom: Cyanotoxin (microcystin) transfer from land to sea otters. PLoS One.

[185] Quiblier C., Wood S., Echenique-Subiabre I., Heath M., Villeneuve A., Humbert J.-F. (2013). A review of current knowledge on toxic benthic freshwater cyanobacteria—ecology, toxin production and risk management. Water Res.

[186] Kudela R.M. (2011). Characterization and deployment of Solid Phase Adsorption Toxin Tracking (SPATT) resin for monitoring of microcystins in fresh and saltwater. Harmful Algae.

[187] Backer L.C., Grindem C.B., Corbett W.T., Cullins L., Hunter J.L. (2001). Pet dogs as sentinels for environmental contamination. Sci. Total Environ..

[188] Paerl H.W., Huisman J. (2008). Blooms like it hot. Science.

[189] Paerl H.W., Huisman J. (2009). Climate change: A catalyst for global expansion of harmful cyanobacterial blooms. Environ. Microbiol. Rep..

[190] DogHeirsTeam (2011). Toxic Blue-Green Algae in Luna Pier. NeighborHound Watch.

[191] NCCOS Phytoplankton Monitoring Network homepage. http://products.coastalscience.noaa.gov/pmn/.

[192] Glynn M., Backer L., Lee L., Teutsch S., Thacker S., St. Louis M. (2010). Collecting Public Health Surveillance Data: Creating a Surveillance System. Principles and Practice of Public Health Surveillance.

